# Bacterial vaginosis, vulvovaginal candidiasis and trichomonal vaginitis among reproductive-aged women seeking primary healthcare in Sana’a city, Yemen

**DOI:** 10.1186/s12879-019-4549-3

**Published:** 2019-10-22

**Authors:** Maha Abdul-Aziz, Mohammed A. K. Mahdy, Rashad Abdul-Ghani, Nuha A. Alhilali, Leena K. A. Al-Mujahed, Salma A. Alabsi, Fatima A. M. Al-Shawish, Noura J. M. Alsarari, Wala Bamashmos, Shahad J. H. Abdulwali, Mahdi Al Karawani, Abdullah A. Almikhlafy

**Affiliations:** 1 Department of Obstetrics and Gynecology, Faculty of Medicine, University of Science and Technology, Sana’a, Yemen; 2 Tropical Disease Research Center, Faculty of Medicine, University of Science and Technology, Sana’a, Yemen; 3 Department of Parasitology, Faculty of Medicine, Sana’a University, Sana’a, Yemen; 4 Medical Laboratory Sciences Program, Faculty of Medicine, University of Science and Technology, Sana’a, Yemen; 5 Department of Community Medicine, Faculty of Medicine, University of Science and Technology, Sana’a, Yemen

**Keywords:** Bacterial vaginosis, Vulvovaginal candidiasis, Trichomonal vaginitis, Vaginitis, Reproductive-aged women, Yemen

## Abstract

**Background:**

In Yemen, the underlying causes of infectious vaginitis have been neglected. Therefore, this study aimed to determine the prevalence and risk factors associated with bacterial vaginosis (BV), vulvovaginal candidiasis (VVC) and trichomonal vaginitis (TV) among non-pregnant reproductive-aged women.

**Methods:**

A cross-sectional study was conducted among 347 non-pregnant reproductive-aged women seeking primary healthcare in Sana’a city, Yemen. Data about sociodemographic characteristics, lifestyle-related behaviors, routine hygienic practices, menstrual care and history and type of contraceptive intake were collected using a structured questionnaire. Vaginal discharge samples were collected and examined for discharge characteristics and pH by a gynecologist. Then, samples were examined for BV, VVC and TV. Data were analyzed using suitable statistical tests.

**Results:**

Vaginal infections were prevalent among 37.6% of reproductive-aged women, where BV was the most prevalent (27.2%). VVC was significantly higher among symptomatic women and significantly associated with itching (*P* = 0.005). Using bivariate analysis, the age of < 25 years (odds ratio [OR] = 1.9, 95% confidence interval [CI]: 1.16–3.10; *P* = 0.010) and using intrauterine contraceptive devices (IUCDs) (OR = 1.8, 95% CI: 1.09–2.89; *P* = 0.020) were significantly associated with BV, while history of miscarriage was significantly associated with a lower risk of BV (OR = 0.5, 95% CI: 0.31–0.85, *P* = 0.009). However, polygyny was significantly associated with VVC (OR = 3.4, 95% CI: 1.33–8.66; *P* = 0.007). Multivariable analysis confirmed that age of < 25 years and using IUCD were the independent predictors of BV, while history of miscarriage was an independent protective factor against BV. On the other hand, marriage to a polygamous husband was the independent predictor of VVC.

**Conclusions:**

More than a third of non-pregnant reproductive-aged women seeking PHC in Sana’a have single or mixed infections with BV, VVC or TV. BV is the most frequent cause of vaginitis and is significantly associated with the age of < 25 years and using IUCDs, while VVC is significantly higher among women with polygamous husbands. Health education of polygamous husbands and their wives, regular monitoring of BV among IUCD users and screening women for vaginitis before treatment are recommended**.**

## Background

Inflammation of the vagina, or vaginitis, is caused by various infectious and non-infectious factors [1]. The most common causes of infectious vaginitis are bacterial vaginosis (BV), vulvovaginal candidiasis (VVC) and trichomonal vaginitis (TV) [2]. The healthy vaginal tract of reproductive-aged women is colonized by normal microbiota dominated by lactobacilli, which protect against pathogenic bacterial species when present in sufficient numbers [3]. Therefore, depletion of lactobacilli distorts the balance of the vaginal microbiota and leads to an increase in anaerobic organisms*,* contributing to BV [4, 5]. Although BV is most commonly asymptomatic, it can be characterized by the discharge of homogeneous grayish-white smelly secretions, fishy smell after intercourse or during menstruation and an elevation of vaginal pH to above 4.5 [6–9]. The prevalence of BV ranges from 8 to 51%, depending on geographical location, socioeconomic status and ethnicity [10].

VVC is caused by the overgrowth of yeasts, mainly *Candida albicans*, which are essentially part of the vaginal flora [11]. Symptoms of VVC include vaginal discharge, itching, pain, and swelling. In addition, vulvar erythema and edema with excoriations are common findings. The typical vaginal discharge in VVC is described as cottage cheese-like in character [9]. It has been suggested that 75.0% of women may experience VVC during their lifetimes [12].

*Trichomonas vaginalis* is a flagellate protozoan parasite that causes trichomoniasis, which is mainly characterized by severe vaginitis among symptomatic females. The global incidence of trichomoniasis cases was estimated at 140.8 million [95% Uncertainty interval (UI): 121.2–163.2 million] in 2015, with a percentage change of 15.4 (14.5 to 16.5) between 2005 and 2015 [13]. Its transmission is usually sexual, and frequent recurrences often occur if the male partner is not simultaneously treated. Women with TV may complain of yellowish-green, foul-smelling, frothy vaginal discharge. Additionally, dysuria, dyspareunia, vulvar itching and pain may be found. The vulva may be erythematous, edematous and excoriated, and subepithelial hemorrhages or “strawberry spots” may be observed on the vagina and cervix [9].

Vaginitis has been associated with serious sequelae. BV during pregnancy increases the risk of preterm birth and miscarriage [14, 15]. TV can increase the transmission of human immunodeficiency virus [16], while VVC during pregnancy may lead to preterm birth [17]. Over the past 10 years, several risk factors of vaginitis have been identified. Douching, multiple partners and intrauterine contraceptive devices (IUCDs) are risk factors of BV [18–21], while low socioeconomic status, low educational level, douching and poverty are related to TV [22], and immunodeficiency, diabetes and recent antibiotic use are risk factors of VVC [23–25].

In Yemen, vaginitis is one of the most common conditions behind seeking medical care (Personal communication, M. Alhaj, 2019). Recently, BV has been reported among 39.2% of pregnant women in Hadhramout governorate, east of Yemen [26], while TV has been reported among 11.1% of pregnant women seeking primary healthcare (PHC) in Sana’a city [27]. Yet, the prevalence and risk factors associated with the infectious causes of vaginitis among reproductive-aged women are still unclear. Therefore, the present study aimed to determine the prevalence and risk factors associated with the most common infectious causes of vaginitis among Yemeni women.

## Methods

### Study design, area and population

A cross-sectional study was conducted among reproductive-aged women seeking healthcare in PHC centers in Sana’a city, the capital of Yemen, in the period from February to December 2017. Women were excluded from participation if they were menstruating, pregnant, or if they had received antibiotic or antifungal therapies in the preceding week or vaginal douching within the previous 24 h.

### Sample size and sampling strategy

Cluster sampling was adopted, where all PHC centers in Sana’a were listed and four centers were randomly selected. Then, all reproductive-aged women attending each center were invited to voluntarily participate until obtaining the sample size required. The minimum sample size calculated was 294 women at a 95% confidence interval (CI), a precision of 7.0%, an expected prevalence rate of 50.0% and a design effect of 1.5. Yet, 347 women were included in the study.

### Data collection

Data about sociodemographic characteristics, lifestyle-related behaviors, routine hygienic practices, menstrual care and history of contraceptive intake were collected using a structured questionnaire through face-to-face interview.

### Vaginal examination

The vagina of each woman was examined by a gynecologist for the characteristics of vaginal discharge (color, consistency and odor) using a dry sterile speculum. Then, vaginal pH was measured by applying a pH paper to its lateral wall.

### Laboratory investigations

Clinical samples were collected from vaginal walls with two cotton-tipped swabs. The vaginal swabs were then inoculated into a tube containing approximately 2 ml of saline and transported to the Microbiology Laboratory of the University of Science and Technology Hospital. Gram-stained smears were prepared, examined and interpreted for the diagnosis of BV according to the Nugent scoring system [28]. A score of ≥7 was interpreted as positive for BV [29]. Ten percent potassium hydroxide (KOH) wet mounts were examined for *C. albicans* yeasts or pseudohyphae followed by colony identification after cultivation on Sabouraud dextrose agar for the diagnosis of VVC [30]. Saline wet mounts were examined for motile trophozoites of *T. vaginalis* followed by their morphological identification on Giemsa-stained smears for the diagnosis of TV [31].

### Statistical analysis

Data were analyzed using IBM SPSS Statistics for Windows, version 23.0 (IBM Corp., Armonk, NY, USA). Frequencies and proportions were used to summarize and present the data. The association between independent and dependent variables was tested using Pearson’s chi-square or Fisher’s exact test, whichever suitable, and the odds ratio (OR) and its corresponding 95% CI were reported. A Multivariable logistic regression model was developed for all variables included in the bivariate analysis, and the adjusted OR and its corresponding 95% CI were reported. *P*-values < 0.05 were considered statistically significant.

## Results

### Characteristics of the study population

The age of reproductive-aged women in the present study ranged from 15 to 50 years, with a median age of 28.0 years (interquartile range: 10). Of 347 women, the majority of women were aged between 26 and 35 years (46.5%), of secondary level of education (38.6%), married (98.3%), urban residents (89.3%) and unemployed (90.5%). Approximately half of the women were living in rented houses (Table 1).

**Table 1:** Characteristics of reproductive-aged women attending PHC centers in Sana’a city, Yemen (2017)^a^

Characteristic	*n* (%)
Age (years)	
< 26	123 (35.5)
26–35	161 (46.5)
> 35	62 (17.9)
Education level	
Illiterate	70 (20.2)
University or above	64 (18.4)
Secondary	134 (38.6)
Primary	79 (22.8)
Marital status	
Married	341 (98.30)
Divorced or widow	6 (1.7)
Residence	
Urban	310 (89.3)
Rural	37 (10.7)
Employment status	
Employed	33 (9.50)
Unemployed	314 (90.5)
Ownership of a house	
Yes	168 (48.4)
No	179 (51.6)

^a^The total women included in the study were 347

### Prevalence and association of vaginal infections with symptomatic presentation

Table 2 shows an overall prevalence of 37.6% for any type of vaginal infections among reproductive-aged women. BV was the most frequent single infection (27.2%) followed by VVC among 6.6% of women. In contrast, TV was the least frequent vaginal infection, where only three (0.9%) women were found to be positive. Mixed infection with BV and VVC was observed among 2.6% of women, while mixed infection with TV and VVC was observed among 0.3% of women. Table 3 shows that BV was not significantly associated with the symptomatic presentation, characteristics of vaginal discharge or vulvovaginal itching. In contrast, VVC was significantly higher among symptomatic women (*P* = 0.006) and significantly associated with vulvovaginal itching (*P* = 0.005).

**Table 2:** Prevalence of vaginal infections among reproductive-age women attending PHC centers in Sana’a city, Yemen (2017)^a^

Type of vaginal infection	Prevalence		
	*n*	(%)	95% CI
Overall prevalence (any type)	130	(37.6)	(32.5–42.7)
Single infections			
BV	94	(27.2)	(22.7–32.0)
VVC	23	(6.6)	(4.5–9.7)
TV	3	(0.9)	(0.3–2.5)
Mixed infection			
BV and VVC	9	(2.6)	(1.4–4.9)
TV and VVC	1	(0.3)	(0.1–1.6)

^a^The total women included in the study were 347; *n*, number of infected women, *CI* confidence interval, *BV* bacterial vaginosis, *VVC* vulvovaginal candidiasis, *TV* trichomonal vaginitis

**Table 3:** Association of vaginal infections with certain clinical features among reproductive-aged women attending PHC centers in Sana’a, Yemen (2017)

Feature	*N*	BV		VVC	
		*n* (%)	*P*-value	*n* (%)	*P*-value
Symptomatic presentation					
No	86	23 (26.7)	0.479	2 (2.3)	0.006*
Yes	260	80 (30.8)		31 (11.9)	
Color of discharge					
Clear to white	279	81 (29.0)	0.541	26 (9.3)	0.778
Grey-yellow, milky or brown	67	22 (32.8)		7 (10.4)	
Consistency of discharge					
Watery and scanty	141	37 (26.2)	0.234	9 (6.4)	0.980
Thick and profuse	205	66 (32.2)		24 (11.7)	
Odor of discharge					
Non-offensive	245	67 (27.3)	0.125	23 (9.4)	0.883
Unpleasant	101	36 (35.6)		10 (9.9)	
Vulvovaginal itching					
Yes	96	31 (32.3)	0.525	16 (16.7)	0.005
No	250	72 (28.8)		17 (6.8)	

*N* number of women examined, *n* number of women positive for BV or VVC, *BV* bacterial vaginosis, *VVC* vulvovaginal candidiasis, ^*^Fisher’s exact test was used

Association of certain sociodemographic factors, women’s practices and history of poor obstetric outcomes with vaginal infections among reproductive-aged women.

Bivariate analysis showed that women aged < 25 years (OR = 1.9, 95% CI: 1.16–3.10; *P* = 0.010) were at about two times higher risk of BV. In contrast, education, residence, employment status, husband’s employment status, polygyny and being married for the first time were not significantly associated with BV. On the other hand, polygyny was the only sociodemographic factor significantly associated with VVC, where those married to polygamous husbands were at about three and half times higher risk of being infected with VVC than those married to monogamous husbands (OR = 3.4, 95% CI: 1.33–8.66; *P* = 0.007). Although women married for more than once were 2.8 times more likely to be infected with VVC (OR = 2.8, 95% CI: 0.96–7.97 *P* = 0.007; *P* = 0.051), the significance of the association was on the borderline (Table 4).

**Table 4:** Bivariate analysis of the association of sociodemographic factors with BV and VVC among women attending the PHC centers in Sana’a city, Yemen (2017)

Variable	*N*	BV			VVC		
		*n* (%)	OR (95% CI)	*P*-value	*n* (%)	OR (95% CI)	*P*-value
Age (years)							
≥ 25	245	63 (25.7)	Reference		23 (9.4)	Reference	
< 25	101	40 (39.6)	1.9 (1.16–3.10)	0.010	10 (9.9)	1.1 (0.49–2.32)	0.883
Education							
University	64	15 (23.4)	Reference		5 (7.8)	Reference	
Pre-university	212	62 (29.2)	1.4 (0.71–2.59)	0.366	21 (9.9)	1.3 (0.47–3.59)	0.616
Not educated	70	26 (37.1)	1.9 (0.91–0.91)	0.088	7 (10.0)	1.3 (0.39–4.36)	0.659
Residence							
Urban	309	91 (29.4)	Reference		30 (9.7)	Reference	0.754
Rural	37	12 (32.4)	1.2 (0.55–2.39)	0.708	3 (8.1)	0.8 (0.24–2.83)	
Employment status							
Employed	33	8 (24.2)	Reference		3 (9.1)	Reference	0.927
Housewife	313	95 (30.4)	1.4 (0.59–3.13)	0.465	30 (9.6)	1.1 (0.31–3.68)	
Husband’s employment status							
Employed	284	85 (29.9)	Reference		26 (9.2)	Reference	0.604
Unemployed	62	18 (29.0)	1.0 (0.52–1.75)	0.889	7 (11.3)	1.3 (0.52–3.06)	
Polygyny							
No	316	93 (29.4)	Reference		26 (8.2)	Reference	0.007
Yes	30	10 (33.3)	1.2 (0.54–2.66)	0.655	7 (23.3)	3.4 (1.33–8.66)	
First marriage							
Yes	322	95 (29.5)	Reference		28 (8.7)	Reference	0.051
No	24	8 (33.3)	1.2 (0.49–2.89)	0.692	5 (20.8)	2.8 (0.96–7.97)	

*N* number of examined women, *n* number of infected women, *BV* bacterial vaginosis, *VVC* vulvovaginal candidiasis, *OR* odds ratio, *CI* confidence interval, *IUCD* intrauterine contraceptive device

Table 5 shows that using IUCD was the only practice significantly associated with BV among reproductive-aged women, where users were about two times more likely to be infected compared with their counterparts (OR = 1.8, 95% CI: 1.09–2.89; *P* = 0.020). In contrast, history of miscarriage was significantly associated with a half lower risk of BV among women (OR = 0.5, 95% CI: 0.31–0.85, *P* = 0.009). Multivariable analysis of the sociodemographic factors, practices and poor obstetric outcomes identified the age of < 25 years (AOR = 2.0, 95%CI: 1.10–3.62; *P* = 0.023) and using IUCD (AOR = 1.8, 95% CI: 1.04–3.08; *P* = 0.036) as the independent predictors of BV. However, history of miscarriage was identified as an independent protective factor (AOR = 0.5, 95% CI: 0.26–0.81; *P* = 0.006) against BV among reproductive-aged women seeking PHC in Sana’a. On the other hand, marriage to a polygamous husband was identified as an independent predictor of VVC, where women with polygamous husbands were four times more likely to get infected (AOR = 3.9, 95% CI: 1.15–13.29; *P* = 0.029) (Table 6).

**Table 5:** Bivariate analysis of the association of the practices and history of poor obstetric outcomes with BV and VVC among women attending the PHC centers in Sana’a city, Yemen (2017)

Variable	*N*	BV			VVC		
		*n* (%)	OR (95% CI)	*P*-value	*n* (%)	OR (95% CI)	*P*-value
Using IUCD							
No	242	63 (26.0)	Reference	0.020	21 (8.7)	Reference	0.406
Yes	104	40 (38.5)	1.8 (1.09–2.89)		12 (11.5)	1.4 (0.65–2.91)	
Using local antibiotics							
No	148	48 (32.4)	Reference	0.349	14 (9.5)	Reference	0.966
Yes	198	55 (27.8)	0.8 (0.50–1.27)		19 (9.6)	1.0 (0.49–2.09)	
Using systemic antibiotics							
No	181	57 (31.5)	Reference	0.463	13 (7.2)	Reference	0.118
Yes	165	46 (27.9)	0.8 (0.53–1.34)		20 (12.1)	1. 8 (0.86–3.71)	
Smoking							
No	276	76 (27.5)	Reference	0.071	26 (9.4)	Reference	0.883
Yes	70	27 (38.6)	1.7 (0.95–2.86)		7 (10.0)	1.1 (0.44–2.57)	
History of miscarriage							
No	216	75 (34.7)	Reference	0.009	23 (10.6)	Reference	0.365
Yes	130	28 (21.5)	0.5 (0.31–0.85)		10 (7.7)	0.7 (0.32–1.52)	
History of preterm labor							
No	311	89 (28.6)	Reference	0.163	28 (9.0)	Reference	0.313
Yes	35	14 (40.0)	1.7 (0.81–3.42)		5 (14.3)	1.7 (0.61–4.69)	
Using sanitary napkins							
Yes	301	90 (29.9)	Reference	0.890	29 (9.6)	Reference	0.874
No	45	13 (28.9)	1.0 (0.48–1.89)		4 (8.9)	0.9 (0.31–2.74)	
Regular vaginal douching							
Yes	111	27 (24.3)	Reference	0.128	9 (8.1)	Reference	0.534
No	235	76 (32.3)	1.5 (0.89–2.48)		24 (10.2)	1.3 (0.58–2.87)	
Drying genital area							
Yes	130	32 (24.6)	Reference	0.104	9 (6.9)	Reference	0.199
No	216	71 (32.9)	1.5 (0.92–2.45)		24 (11.1)	1.7 (0.76–3.74)	
Privacy of toilet							
Personal	96	33 (34.4)	Reference	0.246	9 (9.4)	Reference	0.949
Shared	250	70 (28.0)	0.7 (0.45–1.23)		24 (9.6)	1.0 (0.46–2.29)	
Toilet type							
Arabic	331	96 (29.0)	Reference	0.143	23 (10.1)	Reference	0.699
European	15	7 (46.7)	2.1 (0.76–6.07)		10 (8.4)	0.8 (0.37–1.77)	
Preferred clothing							
Wide clothes	279	84 (30.1)	Reference	0.779	28 (10.0)	Reference	0.520
Tight clothes	67	19 (28.4)	0.9 (0.51–1.66)		5 (7.5)	0.7 (0.27–1.95)	
Method of genital area cleaning							
Forward	212	60 (28.3)	Reference	0.453	23 (10.8)	Reference	0.296
Backward	134	60 (32.1)	1.2 (0.75–1.92)		10 (7.5)	0.7 (0.31–1.44)	

*N* number of examined women, *n* number of infected women, *BV* bacterial vaginosis, *VVC* vulvovaginal candidiasis, *OR* odds ratio, *CI* confidence interval, *IUCD* intrauterine contraceptive device

**Table 6:** Independent predictors of BV and VVC among women attending the PHC centers in Sana’a city, Yemen as revealed by multivariable analysis (2017)

Variable	AOR (95% CI)	*P*-value
Independent predictors associated with BV		
Age younger than 25 years	2.0 (1.10–3.62)	0.023
Using IUCD	1.8 (1.04–3.08)	0.036
History of miscarriage	0.5 (0.26–0.81)	0.006
Independent predictor associated with VVC		
Polygyny	3.9 (1.15–13.29)	0.029

*BV* bacterial vaginosis, *VVC* vulvovaginal candidiasis, *AOR* adjusted odds ratio, *CI* confidence interval, *IUCD* intrauterine contraceptive device

## Discussion

The present study revealed that 37.6% of Yemeni reproductive-aged women seeking PHC in Sana’a city have single or mixed vaginal infections with BV, VVC or TV. Such prevalence is almost comparable to those reported among women seeking medical care from Pakistan and Nepal, being 33.5 and 39.0%, respectively [32, 33]. On the contrary, it is lower than the prevalence (89.0%) reported among non-pregnant reproductive-aged women from Rajasthan in India but higher than the prevalence (15.4%) reported among Ethiopian reproductive-aged women seeking medical care [34, 35]. BV was the most common cause of vaginitis among Yemeni women seeking PHC in Sana’a, being more predominant than VVC. However, TV was the least frequent cause of vaginitis, being detected among less than 1.0% of women. The predominance of BV over the other two causes of vaginitis is consistent with the findings among reproductive-aged women from distantly separated countries worldwide, including Indonesia, southwestern Nigeria, Nepal, Iran, Turkey and Grenada [18, 25, 33, 36–38]. In contrast, VVC was the most prevalent cause of vaginitis among sexually active adolescents from Brazil [21] and reproductive-aged women seeking medical care in Ethiopia and northeastern/northwestern Nigeria [35, 39, 40].

Based on the Nugent scoring system as the “gold standard” for BV diagnosis [41], BV was found to be prevalent among 27.2% of Yemeni reproductive-aged women in the present study which is lower than that (39.2%) reported among pregnant women from Hadhramout, an eastern Yemeni governorate [26]. Compared to the finding of the present study, a similar prevalence of 27.0% was reported for BV among women from socioeconomically deprived communities in Peru [42]. However, lower BV prevalence of 15.2 and 19.5% were reported among reproductive-aged women attending hospitals in Ethiopia [35, 38], and non-pregnant women attending PHC centers in Iran (16.2%) [36]. Besides, higher prevalence of 48.6% was reported for BV among women with vaginitis attending hospital in Kochi, India [43]. It is noteworthy that the comparison between studies is difficult due to differences in study designs and populations, diagnostic techniques and technical variations. In addition to the negative impact of BV on the quality of life of Yemeni reproductive-aged women, BV could contribute to infertility [44, 45]. Given the high prevalence of BV among Yemeni women, screening or treatment of reproductive-aged women for BV should be undertaken to avoid its negative health impacts, particularly for women complaining of and seeking treatment for infertility.

The significant association of VVC with vaginal itching among reproductive-aged women in the present study is consistent with a review evaluating vaginal complaints, which suggests more likelihood of vaginal itching among patients with candidiasis [46]. Furthermore, the lack of a significant difference between BV among asymptomatic and symptomatic women is a common observation [47]. It is noteworthy that even asymptomatic BV can contribute to a range of adverse outcomes [10, 48]. This, in turn, supports the need for screening reproductive-aged women irrespective of the symptomatic nature of vaginitis. The significant association between BV and young age is consistent with previous studies elsewhere [49, 50]. The risk of BV in younger women might be explained by the higher frequency of unprotected sexual intercourse among newly married adolescent and young ladies that can affect the vaginal environment in a way that increases the likelihood of BV [51]. This could be supported by the few women reporting the use of condoms (1.2%) in the present study. Frequent sexual intercourse prevents the restoration of the vaginal ecosystem after a coital act and, hence, sustains an ideal environment for the growth of anaerobic bacteria [52]. Frequent sexual intercourse can also increase the likelihood of transferring perianal and perivulvar bacteria to the vagina, leading to BV [53].

The identification of using IUCD as an independent predictor of BV among reproductive-aged women is in line with previous findings from Indonesia and Turkey [18, 54], which reported a significant association between long-term use of IUCD and BV. The significant association of IUCD with BV in the present study also agrees with the findings of earlier studies in Belgium and Sweden [55, 56]. In contrast, an earlier review on the association of IUCD and pelvic inflammatory diseases found no association between IUCD use and BV due to the lack of strong evidence [57]. According to the review, the lack of adequate adjustment for sexual behaviors and the use of inappropriate control groups were among the factors making the evidence of association not convincing [57]. The association between IUCD use and BV in the present study could be attributed to increased menstrual flow and irregular vaginal bleeding, where these can change the vaginal microbiome and decrease the ratio lactobacilli [18, 58]. In this study, vaginal bleeding was observed in 15.4% (16/104) of women using IUCD. Moreover, the IUCD may facilitate the ascent of cervicovaginal microorganisms into the uterus [59]. It has been suggested that about a half of IUCD users can have at least one episode of BV during the first 24 months [55]. Therefore, there is a need for continual monitoring of BV among women using IUCDs, with replacement if needed.

Although miscarriage has been suggested as a poor obstetric outcome of BV [15], history of miscarriage was a protective factor against BV among women in the present study. This finding could be partially explained by the higher awareness among aborted women of the risk of BV, which was translated into correct practices against BV. It is to be noted that a half of women in the present study were aware of vaginitis as a cause of miscarriage. BV has been suggested as a cause of preterm delivery irrespective of treatment [60]. Although the odds of preterm births were 1.7-fold higher among women with BV in the present study, the association did not attain statistical significance. Moreover, specific bacterial species in BV can be associated with preterm births [61], such an association can be influenced by the predominance of certain bacterial species.

In line with the low prevalence of VVC (6.6%) among reproductive-aged women in the present study, Yemen was identified as a country with the least frequent VVC infections on a global scale by a recent systematic review [62]. The present study, being married to a polygamous husband was an independent predictor of infection with VVC, suggesting that husbands may mechanically circulate this fungal infection among their multiple wives. Although the role of sexual transmission of *Candida* is still controversial, penile colonization with *Candida* has been reported [63–65]. This, in turn, highlights the importance of health education of polygamous husbands about sexually transmitted diseases (STDs). The prevalence of TV (0.9%) among reproductive-aged women in the present study is lower than the prevalence (11%) found among pregnant women attending PHC centers in Sana’a city [27]. The very low prevalence in the present study could be attributed to the low sensitivity of microscopic examination of wet mount preparations and Giemsa-stained smears in detecting all infections with TV [66–68].

The low prevalence of mixed vaginal infections (2.9%) among women in the present study entails the discontinuation of vaginitis management as a mixed infection, considering that this practice is thought to be common among physicians in Yemen (personal communication, Alhaj, M. 2019). Nevertheless, focusing on the management of BV as the most common cause of vaginitis by prescribing antibiotics may lead to the spread of VVC [25]. Therefore, routine examination of vaginal swabs is key to the proper clinical management of vaginitis among women in Yemen.

Despite the suitability of the present cross-sectional design to determine the prevalence of vaginal infections, its value identifying the associated risk factors is limited. Therefore, case-control studies are recommended for a comprehensive analysis of the risk factors associated with vaginal infections among reproductive-aged women in Yemen. On the other hand, relying on microscopic examination for the diagnosis of TV could underestimate the prevalence of the infection, and the use of more sensitive techniques is recommended.

## Conclusions

More than a third of non-pregnant reproductive-aged women seeking PHC in Sana’a city have single or mixed infections with BV, VVC or TV, with BV is the most frequent cause of vaginitis among 27.2% of women. BV is significantly associated with the age of < 25 years and using IUCDs. Second to BV as a cause of vaginitis, VVC is significantly higher among women with polygamous husbands. Health education interventions are recommended to raise women’s awareness of vaginitis and its prevention. In addition, regular monitoring of BV among women using IUCD, educating polygamous husbands and their wives about the transmission and prevention of STDs and screening women for the causes of vaginitis before treatment are recommended.

## Data Availability

The datasets used and/or analysed during the current study available from the corresponding author on reasonable request.

## Abbreviations

AOR: Adjusted odds ratio
BV: Bacterial vaginosis
CI: Confidence interval
IUCD: Intrauterine contraceptive device
OR: Odds ratio
PHC: Primary healthcare
SPSS: Statistical Packages for Social Sciences
STD: Sexually transmitted disease
TV: Trichomonal vaginitis
VVC: Vulvovaginal candidiasis
